# Implementation of Sphinx/Lynx as daily QA equipment for scanned proton and carbon ion beams

**DOI:** 10.1002/acm2.13896

**Published:** 2023-01-27

**Authors:** Loïc Grevillot, Jhonnatan Osorio Moreno, Hermann Fuchs, Ralf Dreindl, Alessio Elia, Marta Bolsa‐Ferruz, Markus Stock, Hugo Palmans

**Affiliations:** ^1^ MedAustron Ion Therapy Center Wiener Neustadt Austria; ^2^ Christian Doppler Laboratory for Medical Radiation Research for Radiation Oncology Medical University of Vienna Wiener Neustadt Austria; ^3^ Department of Radiation Oncology Medical University of Vienna/AKH Vienna Wiener Neustadt Austria; ^4^ Department of Oncology Karl Landsteiner University of Health Sciences Wiener Neustadt Austria; ^5^ National Physical Laboratory Teddington UK

**Keywords:** carbon ion, ion beam, proton, QA, radiation therapy

## Abstract

**Purpose:**

Reporting on the first implementation of a proton dedicated commercial device (IBA Sphinx/Lynx) for daily Quality Assurance (QA) of scanned proton and carbon ion beams.

**Methods:**

Daily QA trendlines over more than 3 years for protons and more than 2 years for carbon ions have been acquired. Key daily QA parameters were reviewed, namely the spot size and position, beam range, Bragg peak width, coincidence (between beam and imaging system isocenters), homogeneity and dose.

**Results:**

The performance of the QA equipment for protons and carbon ions was evaluated. Daily QA trendlines allowed us to detect machine performance drifts and changes. The definition of tolerances and action levels is provided and compared with levels used in the literature.

**Conclusion:**

The device has been successfully implemented for routine daily QA activities in a dual particle therapy facility for more than 2 years. It improved the efficiency of daily QA and provides a comprehensive QA process.

## 1 INTRODUCTION

A variety of dosimetry equipment is available in the field of light ion beam therapy, in particular for facilities equipped with scanned ion beam delivery systems. While commercial companies have been developing dosimetry equipment for proton therapy facilities, less equipment has been developed specifically for carbon ions. A review of existing equipment can be found in Refs. [1, 2]. The method for implementing dosimetry equipment at MedAustron was presented in a previous paper.^3^ Several integrated Quality Assurance (QA) devices were developed recently to fasten daily QA of scanned proton beams.^4^, ^5^, ^6^, ^7^ Since 2016, a commercial device called Sphinx (IBA Dosimetry, Schwarzenbruck, Germany) in combination with a scintillator screen called Lynx (IBA Dosimetry) has been made available to facilitate daily QA. The performance of this combination of equipment for the evaluation of proton range was provided in detail in Ref. [8]. The application of the Sphinx/Lynx for comprehensive daily QA of scanned proton beams was described in Ref. [4]. Even if the characterization of Lynx in carbon ion beams was reported,^9^ the routine clinical usage of Lynx and Sphinx devices for carbon ion QA has, to our best knowledge, not been reported so far. The combination of Sphinx/Lynx was implemented at MedAustron for daily proton and carbon ion QA from May 2018 and June 2019, respectively. This paper intends to report on the implementation and performance of Sphinx/Lynx for daily proton and carbon ion QA, as performed at MedAustron for a horizontal fixed beam line. In the next sections, we will describe the main QA equipment specificities, the daily QA concept and review long‐term QA trendlines. The performances of the equipment for protons and carbon ions will be evaluated and the definition of QA tolerances and action levels will be presented.

## 2 MATERIALS AND METHODS

### 2.1 The MedAustron particle therapy accelerator developments

Descriptions of the facility, the technology used at MedAustron,^10^ as well as the specificities of the beam delivery system^11^, ^12^ were presented elsewhere. Only key beam delivery parameters (range, spot size, position, dose, and homogeneity—described later) will be investigated within this report. In addition, for the purpose of this study, we will focus on the data of the horizontal fixed beam line from irradiation room 2 only, further denoted with IR2H. This has the great advantage of enabling a comparison of all physical and clinical aspects of both particle beam types under the same conditions. The terminology IR2Hp or IR2Hc will be used throughout this article to refer to the proton or carbon ion beams delivered through the IR2H beam line. Treatment started in July 2017 for IR2Hp and in July 2019 for IR2Hc. The Sphinx/Lynx was implemented in May 2018 and June 2019 for IR2Hp and IR2Hc. Over the years, the MedAustron Particle Therapy Accelerator (MAPTA) has undergone numerous performance upgrades, which are relevant for this article. Indeed, it is interesting to correlate QA trendline changes with machine upgrades or major repairs. The beam delivery from MAPTA consists in delivering spills of particles with the same energy or at subsequent energies. The beam intensity, that is, the number of particles delivered per unit of time, is a key parameter influencing the treatment time. The intensity depends on the total amount of particles injected in the synchrotron ring and extraction time (see Ref. [11] for more details about machine parameters). Maximizing the beam intensity is a key to fasten beam delivery. Another way of reducing treatment time is to reduce the dead time between spills, by optimizing machine settings that control the ramp‐up and ramp‐down of the electric current of various magnetic components of the machine. The proton intensity was increased by a factor of ~2 after implementation of the **Upgrade 1**, on the 21st of March 2020. Proton and carbon ion delivery times were also reduced by optimizing inter‐spill dead times, via **Upgrade 2** on the 13th of December 2020. Today, the number of particles per spill is approximately 2 × 10^10^ and 4 × 10^8^ for protons and carbon ions, while the spill lengths are about 10s and 4s, respectively. One of our aims was to study if the changes made in these upgrades led to detectable changes in beam output characteristics.

### 2.2 Sphinx/Lynx description

**FIGURE 1:**
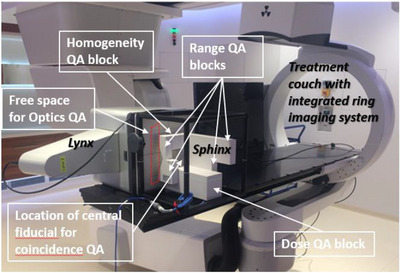
Setup of the equipment (Lynx, Sphinx, ionization chamber) for the measurements in a horizontal beam line.

A detailed discussion of the Sphinx/Lynx system for daily QA of clinical proton beams is available elsewhere^4^ and only a brief overview is provided below. The Sphinx is a modular passive element made of RW3 water‐equivalent plastic material, which is designed to be used in combination with the Lynx detector for 2D relative profile measurements and a plane parallel ionization chamber for dose consistency checks. The Sphinx/Lynx QA system was designed for a 30 × 30 cm^2^ field size. Rigidity and fixation of the system are ensured by a carbon fiber frame. The Sphinx/Lynx equipment contains different regions for QA (Figure 1). Four fixed fiducials are available in the core RW3 block in order to perform image registration. A fifth fiducial is set‐up for testing the coincidence between imaging and beam delivery isocenters. The Lynx detector allows acquiring images (2D maps), which are used to derive dosimetric quantities for QA such as spot sizes, positions, 2D field size, homogeneity, range parameters, etc. The Sphinx/Lynx data acquisition and analysis software is integrated into the *myQA* platform (IBA‐dosimetry). The data acquisition and analysis are managed by the so‐called *Sphinx‐plugin* integrated into the myQA platform and the QA results are automatically saved in the myQA database. For dose QA, ionization chamber readings are saved by the user in an excel file and imported manually in myQA. The full details of the available algorithms are the property of IBA, nevertheless, for sake of clarity, a general description is provided below for the reader. Spot sizes and positions are extracted from a 2D Gaussian fit for each spot. The coincidence test corresponds to the distance between the center of the fiducial and the center of the beam in x and y directions. The QA of the homogeneity is performed by shooting a rectangular mono‐energetic field through a homogeneous RW3 region of 2 cm thickness. The homogeneity (called flatness in myQA) is defined.^13^ and is evaluated in a uniform region as the ratio of S_max_ – S_min_ over S_max_ + S_min_, with S_max_ and S_min_ being the maximum and minimum signal over the uniform region. When an iso‐energetic beam is scanned over an RW3 wedge from the Sphinx, a transversal projection of the longitudinal Bragg peak curve is acquired and can be used for range consistency checks. Different wedge thicknesses are provided to QA ranges of different energies. Depth‐dose profiles are characterized by three independent quantities: the “distal” range (distal depth where the percentage depth dose is 80%), the “proximal” range (proximal depth where the percentage depth dose is 80%) and the fall‐off (distance between distal 80% and 20% dose levels). A fourth quantity can be derived: the width (difference between distal and proximal ranges). These parameters are derived by myQA from projections on the Lynx device and therefore are different from depth‐dose parameters in water in reference conditions. The dose output consistency is checked in a homogeneous RW3 block and acquired at 1 cm depth.

### 2.3 Daily QA set‐up and workflow

The daily QA Sphinx/Lynx setup on the robotic patient positioner for the horizontal fixed beam lines is presented in Figure 1. It includes the four range wedges, a homogeneity region, a coincidence region, spot regions and the dose block in which an Advanced Markus ionization chamber (PTW, Freiburg) is inserted. As the maximum field size at MedAustron is 20 × 20 cm^2^ (while the Sphinx/Lynx QA system was designed for up to 30 × 30 cm^2^ field size), the daily QA was split into a Lynx QA map (including spots, ranges, homogeneity, and coincidence test) and a dose QA map (defined in Section 2.4). In other words, after delivering the Lynx QA map, the treatment couch is moved upwards so that the dose QA map can be delivered to the dose block. The daily Sphinx/Lynx QA of IR2H includes the following key steps: set‐up the Sphinx/Lynx at the planned position on the couch, Sphinx/Lynx image registration against a reference CT image, movement to the treatment position for Lynx QA map acquisition, delivery/acquisition/analysis of the proton Lynx QA map, movement to the treatment position for dose QA, delivery/acquisition/analysis of the proton dose QA map, movement to the treatment position for Lynx QA map acquisition, delivery/acquisition/analysis of the carbon ion Lynx QA map, movement to the treatment position for dose QA, delivery/acquisition/analysis of the carbon ion dose QA map.

### 2.4 Daily QA maps

**FIGURE 2:**
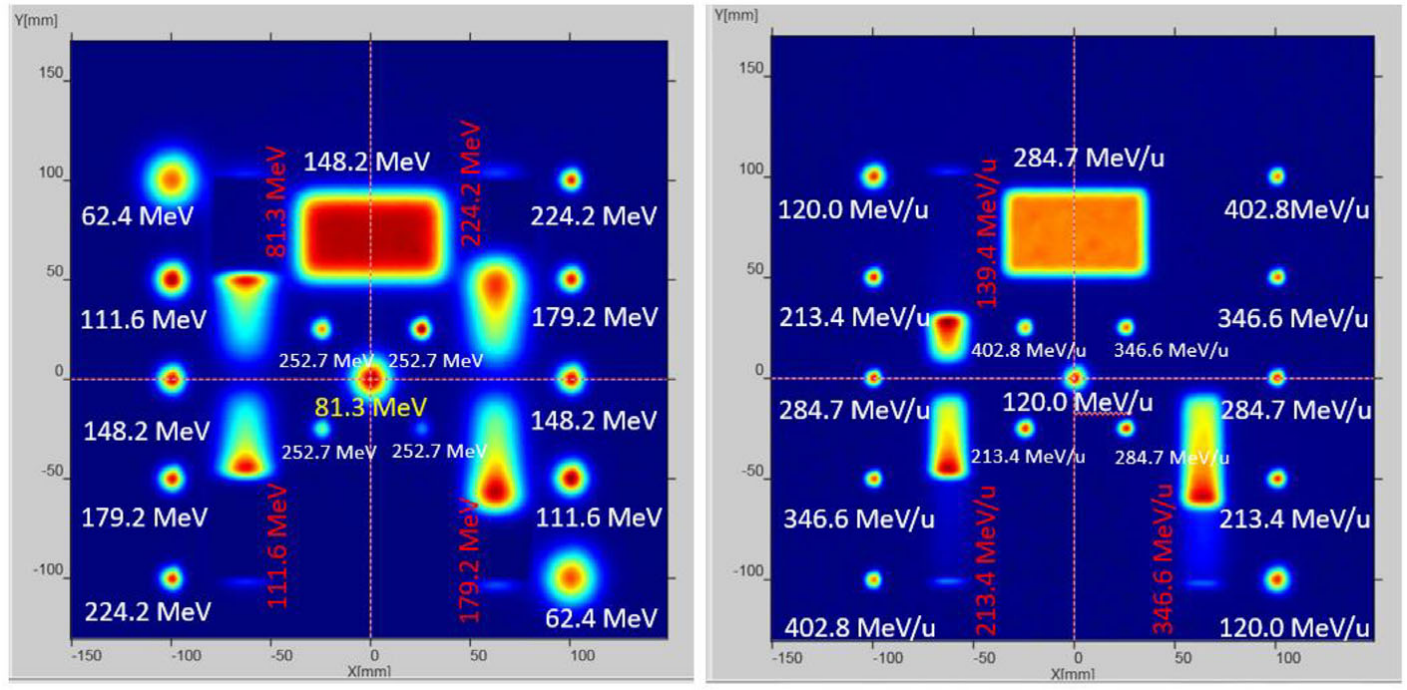
Proton (left) and carbon ion(right) Lynx daily QA maps.

While the number of particles per spill is up to 2 × 10^10^ and 4 × 10^8^ for protons and carbon ions, the maximum number of particles per spot is restricted to 1 × 10^8^ and 2 × 10^6^ during the treatment planning process. For daily QA, we decided to deliver spots with a number of particles similar to the maximum number of particles per spot used for treatment planning. The number of particles per spot for the homogeneity, coincidence, and energy regions were optimized to provide a similar signal intensity in the Lynx image as for the single spots (Figure 2). The proton Lynx QA map was designed for checking spot sizes and positions for 6 nominal beam energies (62.4, 111.6, 148.2, 179.2, 224.2, 252.7 MeV), homogeneity at medium range (148.2 MeV, 7 cm × 4 cm rectangle), reproducibility of four ranges (81.3, 111.6, 179.2, 224.2 MeV) and coincidence at 81.3 MeV. The proton dose QA map allows for checking dose consistency for 4 nominal beam energies (62.4, 97.4, 148.2, 224.2 MeV) in mono‐energetic 6 cm × 6 cm fields at 1 cm depth of RW3. The carbon ion Lynx QA map was designed for checking spot sizes and positions for five nominal beam energies (120, 213.4, 284.7, 346.6, 402.8 MeV/n), homogeneity at medium range (284.7 MeV/n, 7 cm × 4 cm rectangle) and 3 ranges (139.4, 213.4, 346.6 MeV/n). The carbon ion dose QA map allows checking dose consistency for 4 nominal beam energies (120.0, 213.4, 284.7, 346.6 MeV/n) in mono‐energetic 6 cm × 6 cm fields at 1 cm depth of RW3.

### 2.5 Performance of the Sphinx/Lynx QA equipment

The methodology used at MedAustron for acceptance and commissioning of QA equipment was presented earlier^3^ and was followed for the implementation of Sphinx/Lynx. With respect to existing literature, the main purpose of our acceptance and commissioning process was to verify the functionality of the Sphinx/Lynx for the evaluation of carbon ion ranges. The details of the MedAustron commissioning results are out of the scope of this paper. Instead, the evaluation of trendlines provide a unique opportunity to review the performance of the Sphinx/Lynx for proton and carbon ion beams. Trendlines include proton and carbon ion data in the period 15/08/2018–1/12/2021 and 17/06/2019–1/12/2021, respectively. In‐house developed software tools are used to monitor these trendlines on a routine basis.^14^, ^15^ In the context of this study however, the QA data was directly extracted from myQA using the *myQA Cockpit web interface*. Since the same beamline IR2H is used for both particle types, it allows a direct comparison of the performance of the equipment for both particle types.

### 2.6 Definition of QA tolerances and action levels

A tolerance level sets permissible boundary values on the deviation of a quantity from its nominal value. The QA tolerances must be set according to the performances of the QA equipment,^3^ the performances of the beam delivery system (see trendlines from the results section) and clinically acceptable deviations. With respect to performances of the QA equipment or beam delivery system, assuming normal distributions of the statistical fluctuations, setting tolerance levels at 1‐sigma (standard deviation), means that the QA may roughly be out of tolerance every third measurement. It is therefore reasonable to set tolerance levels at 2 sigma level (95% confidence interval). An action level sets boundary values of a quantity beyond which an action has to be taken. Action levels are often set at approximately twice the tolerance level. However, some critical parameters may require tolerance and action levels to be set much closer to each other or even at the same value, to allow detecting machine drifts before reaching clinically acceptable tolerances. According to AAPM TG‐224,^16^ the purpose of a QA program is to provide confidence that the beam delivery is functioning as commissioned for patient treatment and that the planned dose can be delivered safely and accurately within the established tolerance limits. The AAPM TG‐224 provides recommendations on the definition of QA tolerances and periodicity checks for protons. The report is dedicated to proton gantries and encompasses not only scanned beam delivery technique, but also double scattering and uniform scanning. The AAPM TG‐224 is therefore not specific to dual particle facilities and fixed beam lines, as investigated in this article. Nevertheless, we believe it is relevant to compare our tolerances with these recommendations, as they are widely known and used in the proton therapy community.

## 3 RESULTS AND DISCUSSIONS

### 3.1 Trendline analysis for beam range

**FIGURE 3:**
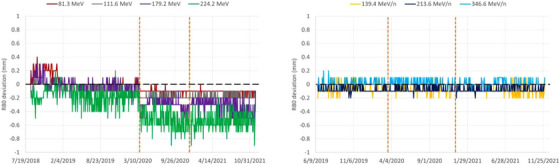
Distal range (R80) Trendlines for protons (left) and carbon ions (right). The two vertical dashed lines mark the introduction of Upgrade 1 and 2, respectively.

**FIGURE 4:**
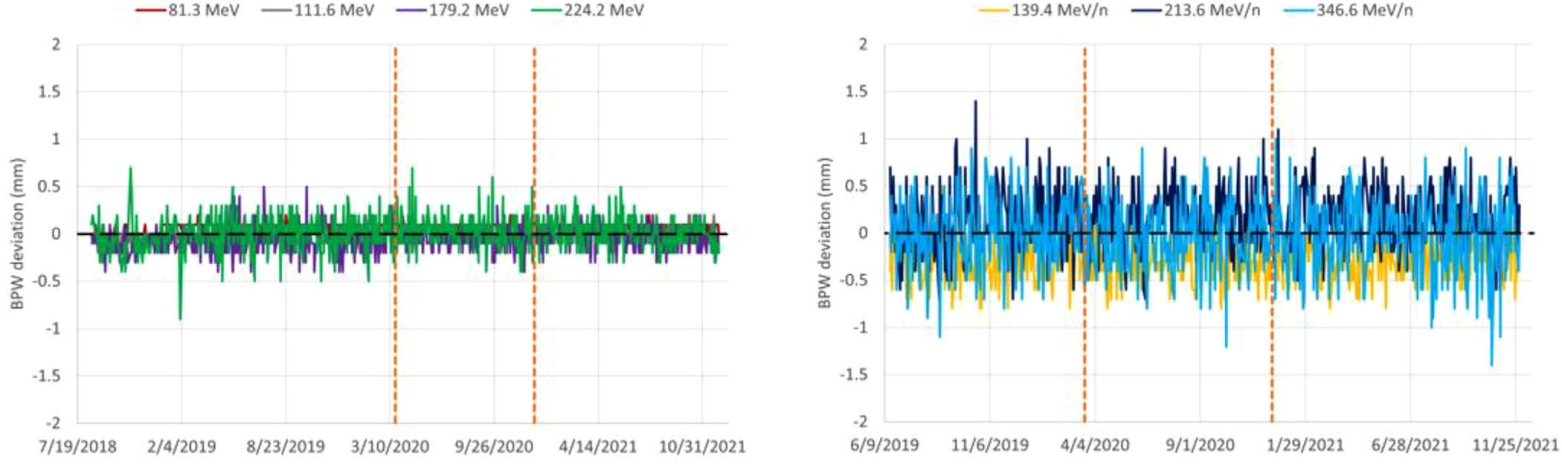
Bragg peak width (BPW) trendlines for protons (left) and carbon ions (right). The two vertical dashed lines mark the introduction of Upgrade 1 and 2, respectively.

The distal range parameter is the key parameter to verify the delivered beam energy (i.e., the mean energy). The carbon ion range trendlines are more stable in terms of statistical fluctuations (standard deviation) than those for protons (Figure 3, Table 1), even if the standard deviations are of the order of 0.1 mm or lower. The proton ranges were reduced after Upgrade 1 and the effect was more pronounced with increasing energy, with up to –0.34 mm at 224.2 MeV (compared to initial values). This effect was also measured during the re‐acceptance of the machine, using range measurements in reference conditions in water and was therefore as expected. In contrast to ranges, Bragg peak width trendlines are more stable for protons than for carbon ions (Figure 4), with standard deviations within 0.0–0.2 mm and 0.2–0.4 mm for protons and carbon ions, respectively (Table 1). This is mostly related to larger uncertainties in the evaluation of the proximal range for carbon ions as compared to protons. This may be partly attributed to the increased quenching effect for carbon ions in Lynx,^9^ leading to a reduced peak‐to‐plateau ratio, as compared to depth‐dose profile measurements in water, and also due to software algorithm limitations related to signal fluctuation in the measurement equipment. The distal fall‐off is the most stable parameter with very small fluctuations (standard deviation always lower than 0.1 mm) for both particle types (Table 1). Even if energy settings can be slightly modified during major machine upgrades, such as with upgrade 1, it is expected that the new energy settings will be stable over time for many years to come. This stability is confirmed over the QA data obtained since treatment start in 2016. Overall, the beam energy parameters delivered by a synchrotron machine are very stable. This statement was confirmed by private communication from colleagues working in other European facilities with similar synchrotron machines.

**TABLE 1:** Deviations of the beam energy parameters from the baseline values and their standard deviations. “Initial machine” means before Upgrade 1, “Upgrade 1” means after Upgrade 1 and before Upgrade 2, “Upgrade 2” means after Upgrade 2

		Distal range (mm)				Fall‐off (mm)				Width (mm)			
	Protons	81.3 MeV	111.6 MeV	179.2 MeV	224.2 MeV	81.3 MeV	111.6 MeV	179.2 MeV	224.2 MeV	81.3 MeV	111.6 MeV	179.2 MeV	224.2 MeV
Initial machine	mean value	0.03	–0.05	0.00	–0.18	0.09	0.01	0.04	–0.01	0.08	–0.02	–0.07	0.01
	std dev	0.08	0.06	0.08	0.11	0.03	0.03	0.05	0.06	0.05	0.08	0.15	0.18
Upgrade 1	mean value	–0.10	–0.18	–0.29	–0.52	0.10	0.01	0.04	0.02	0.09	0.00	–0.05	0.04
	std dev	0.08	0.06	0.08	0.11	0.03	0.03	0.05	0.06	0.05	0.08	0.15	0.18
Upgrade 2	mean value	–0.11	–0.17	–0.33	–0.53	0.10	0.01	0.04	0.03	0.08	–0.01	–0.04	0.02
	std dev	0.04	0.05	0.11	0.12	0.00	0.03	0.05	0.06	0.04	0.07	0.11	0.14

		Distal range (mm)			Fall‐off (mm)			Width (mm)		
	Carbon ions	120.0 MeV/n	284.7 MeV/n	402.8 MeV/n	120.0 MeV/n	284.7 MeV/n	402.8 MeV/n	120.0 MeV/n	284.7 MeV/n	402.8 MeV/n
Initial machine	mean value	–0.08	–0.08	0.02	–0.04	0.01	0.03	–0.29	0.13	0.01
	std dev	0.07	0.05	0.05	0.05	0.03	0.05	0.24	0.36	0.37
Upgrade 1	mean value	–0.10	–0.07	0.03	–0.04	0.01	0.02	–0.30	0.16	–0.05
	std dev	0.05	0.05	0.05	0.05	0.02	0.04	0.23	0.30	0.35
Upgrade 2	mean value	–0.10	–0.07	0.02	–0.03	0.00	0.02	–0.29	0.18	–0.03
	std dev	0.05	0.05	0.04	0.05	0.02	0.04	0.20	0.32	0.40

### 3.2 Trendline analysis for beam position and size

**FIGURE 5:**
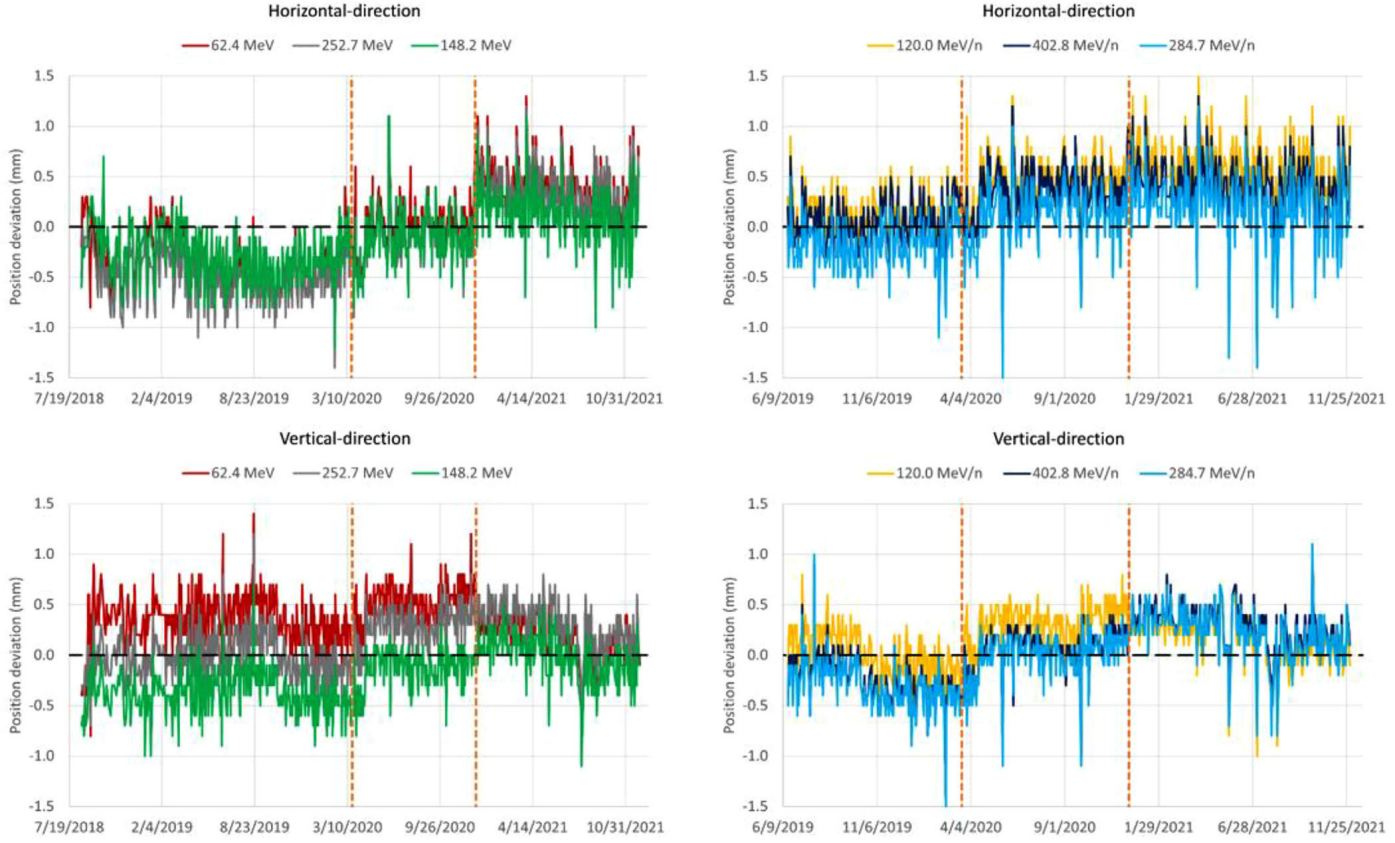
Spot position trendlines for proton (left) and carbon ions (right), in horizontal (first row) and vertical (second row) directions. The two vertical dashed lines mark the introduction of Upgrades 1 and 2.

Deviations of spot positions from the reference (baseline) positions were evaluated for three proton and three carbon ion energies located at different positions over the Sphinx QA map (Figure 5). For protons and carbon ions, the standard deviations of the spot positions (horizontally and vertically) were not significantly affected by the upgrades and were mostly within 0.2–0.3 mm (Table 2). The slightly larger standard deviations in the horizontal direction are visible in Figure 5 and may be related to the beam extraction direction in the synchrotron (which is horizontal). The mean beam positions, however, are dependent on the energy and direction (horizontal or vertical). The most striking improvement is shown for protons in the vertical direction, where the mean beam position energy dependence was corrected after the second machine upgrade (Figure 5). This improvement is not related to Upgrade 2 per se, but results from a major effort to re‐center the beam during the course of Upgrade 2.

**TABLE 2:** Deviations of the spot positions and sizes from the baseline values and their standard deviations for protons (upper table) and carbon ions (lower table), considering the initial machine and the upgraded machine configurations (Upgrades 1 and 2)

		62.4 MeV				148.2 MeV				252.7 MeV			
	Protons	Horizontal	Vertical	Horizontal	Vertical	Horizontal	Vertical	Horizontal	Vertical	Horizontal	Vertical	Horizontal	Vertical
		Pos (mm)	Pos (mm)	FWHM diff	FWHM diff	Pos (mm)	Pos (mm)	FWHM diff	FWHM diff	Pos (mm)	Pos (mm)	FWHM diff	FWHM diff
Initial machine	mean value	–0.33	0.36	–1.2%	–1.3%	–0.31	–0.36	–4.4%	–2.3%	–0.49	0.00	–0.4%	6.0%
	std dev	0.27	0.25	0.3%	0.3%	0.26	0.22	0.4%	1.2%	0.24	0.23	1.2%	1.5%
Upgrade 1	mean value	–0.01	0.52	–3.4%	0.9%	–0.10	–0.13	1.6%	5.1%	–0.13	0.29	4.0%	13.7%
	std dev	0.27	0.20	0.3%	0.4%	0.27	0.19	1.2%	1.6%	0.26	0.19	1.8%	3.0%
Upgrade 2	mean value	0.37	0.21	–3.7%	1.0%	0.11	–0.04	2.1%	6.8%	0.30	0.27	6.5%	16.0%
	std dev	0.27	0.23	0.5%	0.3%	0.27	0.23	0.6%	1.2%	0.27	0.22	1.2%	2.0%

		120.0 MeV/n				284.7 MeV/n				402.8 MeV/n			
	Carbon ions	Horizontal	Vertical	Horizontal	Vertical	Horizontal	Vertical	Horizontal	Vertical	Horizontal	Vertical	Horizontal	Vertical
		Pos (mm)	Pos (mm)	FWHM diff	FWHM diff	Pos (mm)	Pos (mm)	FWHM diff	FWHM diff	Pos (mm)	Pos (mm)	FWHM diff	FWHM diff
Initial machine	mean value	0.14	0.02	–2.6%	–0.4%	–0.18	–0.31	–4.6%	–1.9%	0.04	–0.24	–4.9%	0.4%
	std dev	0.22	0.25	1.7%	1.7%	0.23	0.24	2.0%	2.9%	0.22	0.24	1.7%	3.8%
Upgrade 1	mean value	0.43	0.31	–4.1%	–0.2%	0.13	0.00	–3.7%	–1.4%	0.34	0.05	–4.6%	2.2%
	std dev	0.29	0.22	1.1%	1.0%	0.29	0.22	1.7%	2.4%	0.29	0.22	1.8%	2.5%
Upgrade 2	mean value	0.56	0.15	–3.1%	–2.2%	0.20	0.21	–2.6%	–3.7%	0.38	0.27	–3.5%	2.2%
	std dev	0.32	0.25	1.4%	0.9%	0.32	0.25	1.8%	1.5%	0.31	0.24	1.3%	2.5%

**FIGURE 6:**
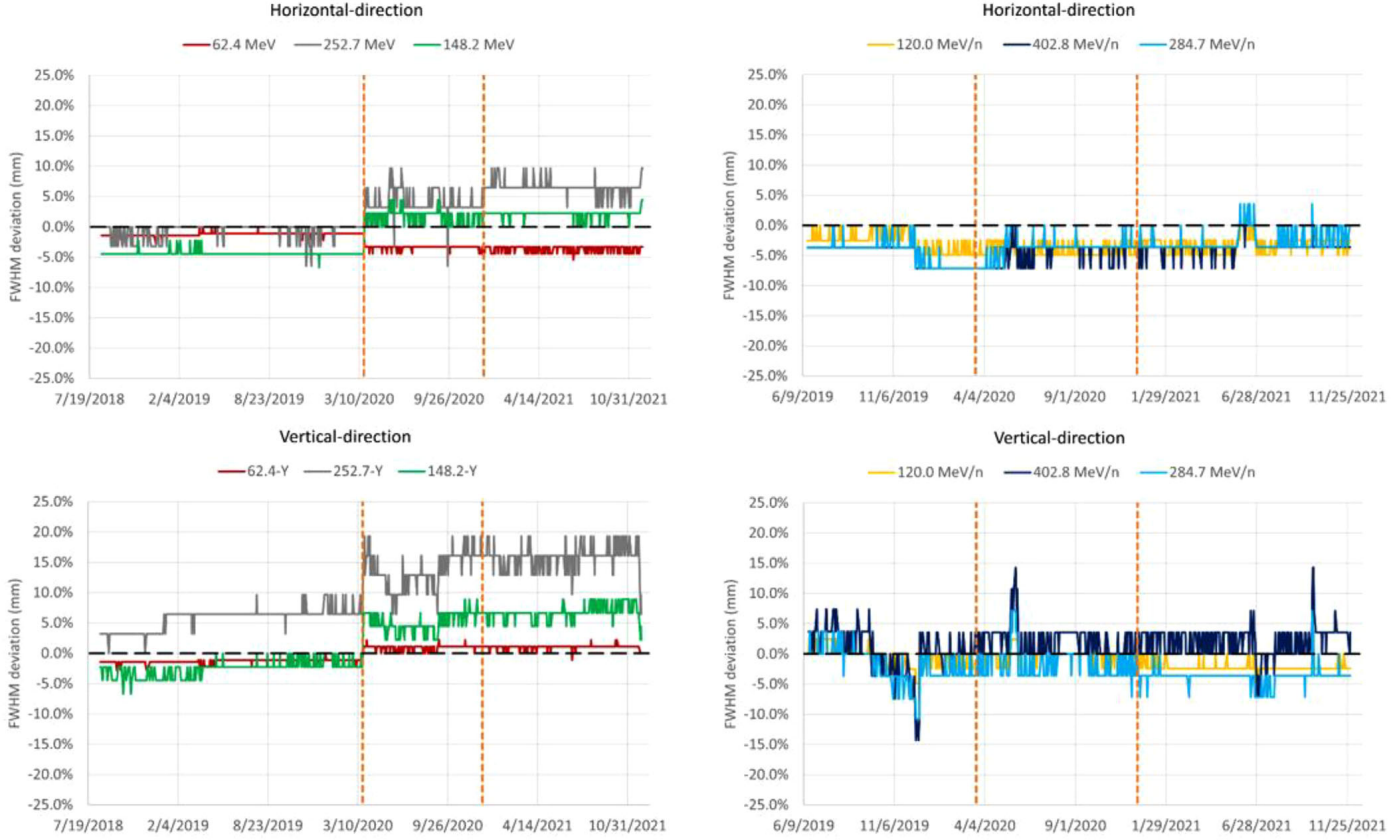
Spot FWHM trendlines for protons (left) and carbon ions (right), in horizontal (first raw) and vertical (second raw) directions. The two vertical dashed lines mark the introduction of Upgrades 1 and 2.

Deviations of the spot sizes (in terms of FWHM) from the reference values measured during commissioning are shown in Figure 6. For carbon ions, Upgrade 2 affected the beam sizes in neither of the two directions (Table 2, Figure 6). For protons, however, the mean spot sizes were significantly altered at each Upgrade (1 and 2), especially as the energy increased. The variations were significantly larger in the vertical direction at medium and high energy, with up to 10.0% spot size increase for the 252.7 MeV proton beam, as compared to the initial value. In contrast, the mean spot size at the lowest proton energy decreased by 2.5% in the horizontal direction, as compared to the initial value. These deviations were accepted, considering the fact that proton spot size increases by approximately a factor 3 at 252.7 MeV until the Bragg peak position: roughly speaking, the 7 mm spot size in air at patient entrance will become approximatively 21 mm at the Bragg peak depth due to multiple Coulomb scattering in the patient over the 38 cm beam range. In addition, one should note that spot sizes in air at phantom entrance and beam widening in water due to multiple Coulomb scattering are added in quadrature and thus spot sizes in water at the Bragg peak depth become dominated by the scattering process in water. Thus, if a 7 mm spot size in air at entrance scatters up to a 21 mm spot size in the Bragg peak in water, an 8.1 mm spot size in air (i.e., 16% increased spot size compared to the initial 7 mm spot size) becomes 21.4 mm in the Bragg peak in water, that is, only 2% larger.

### 3.3 Trendline analysis for coincidence

**FIGURE 7:**
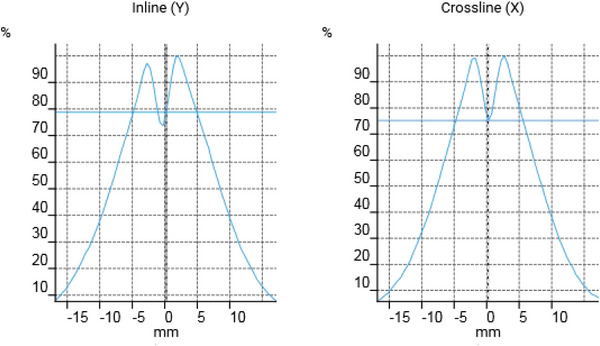
Example of dose profiles extracted from the coincidence test. The dose reduction close to the center of the spot is due to the fiducial.

**FIGURE 8:**
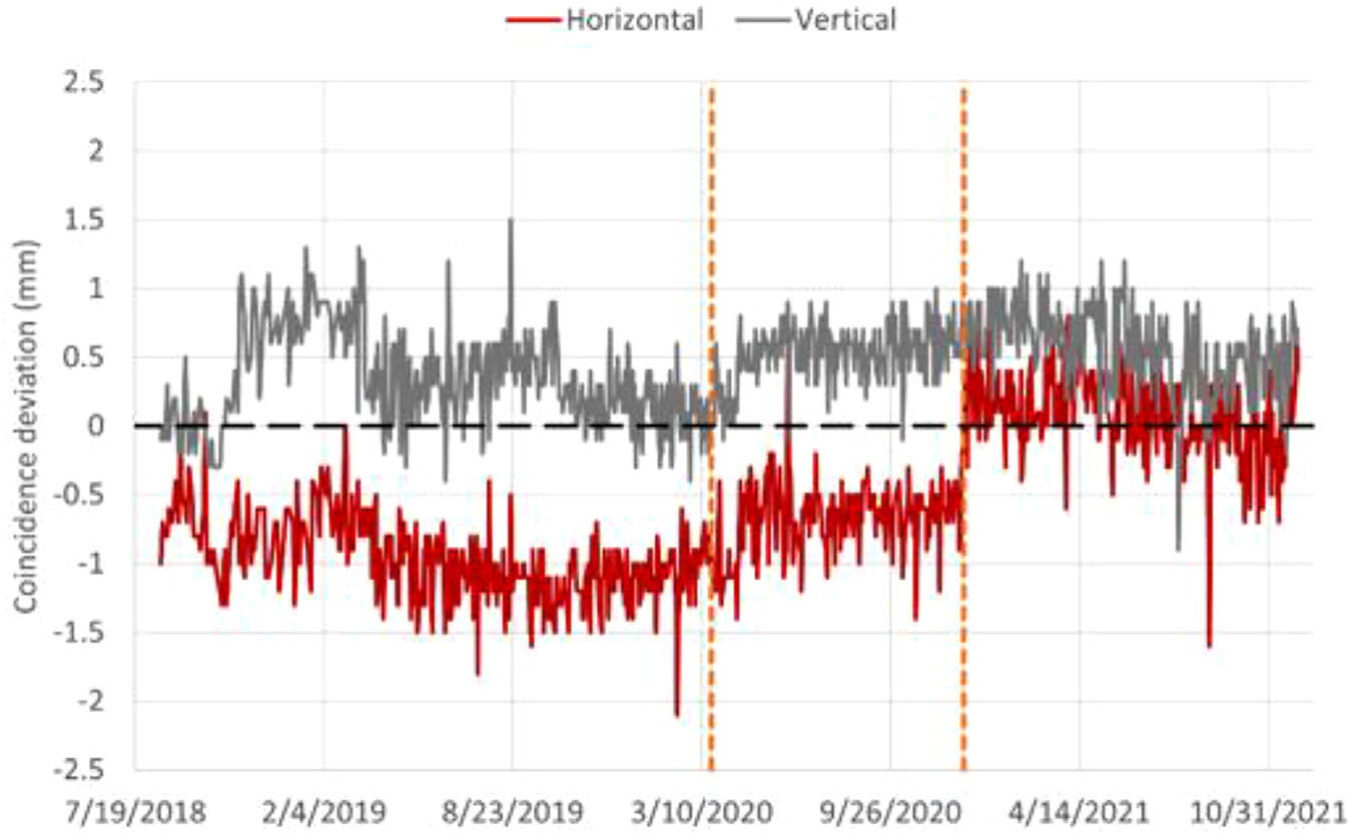
Coincidence trendlines for protons. The two vertical dashed lines mark the introduction of Upgrades 1 and 2.

Trendlines were evaluated for the 81.3 MeV proton beam and an example of the coincidence test result is presented in Figure 7. The improved beam centering in terms of mean beam position after Upgrade 2, as presented earlier, is visible on the coincidence testing trendline (Figure 8). The coincidence test shows the agreement between the imaging isocenter and the beam delivery isocenter. It gives an estimate of how accurate the beam can be delivered to the treatment target after image registration and patient positioning.

### 3.4 Trendline analysis for homogeneity

**FIGURE 9:**
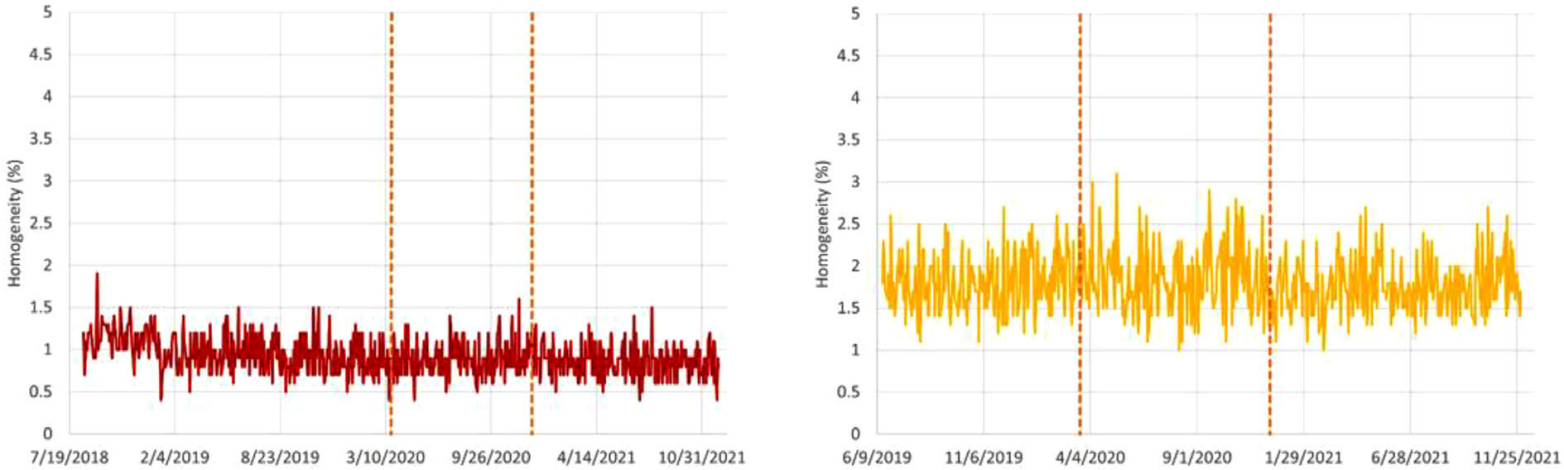
Homogeneity trendlines for protons (left) and carbon ions (right). The two vertical dashed lines mark the introduction of Upgrades 1 and 2.

Trendlines of the homogeneity are presented in Figure 9. The spot sizes are 10.6 and 6.7 mm, for the 148.2 MeV protons and 284.7 MeV/n carbon ions, respectively. The spot spacing was set to roughly 1/3 of the FWHM and are 3 and 2 mm, for protons and carbon ions, respectively. The homogeneity is lower (and therefore better) for protons (0.9% ± 0.2%) than for carbon ions (1.7% ± 0.3%). The mean and standard deviations values remained stable over the course of the two Upgrades, nevertheless the slightly larger standard deviation for carbon ions is visible in Figure 9. This may be explained by several factors: the smaller spot sizes and lower multiple Coulomb scattering for carbon ions as compared to protons, as well as slightly different machine performances for the different particle types.

### 3.5 Trendline analysis for absorbed dose to water

**FIGURE 10:**
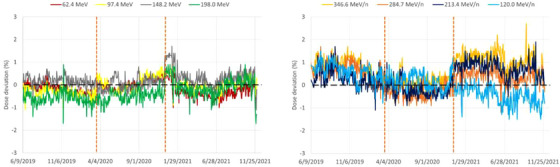
Dose output trendlines for protons (left) and carbon ions (right). The two vertical dashed lines mark the introduction of Upgrades 1 and 2.

Dose readings measured with Sphinx are converted into dose to water in reference conditions (following TRS‐398 protocol) by means of correction factors (established during commissioning as the ratio between the reading in reference conditions and the reading in the sphinx) and compared to reference doses obtained during commissioning (Figure 10 and Table 3). For protons, Upgrade 1 and Upgrade 2 did not have a major effect on the dose and standard deviations were within 0.2%–0.4%. Nevertheless, energy‐dependent dose variations can be observed in terms of mean dose deviations: the 62.4 and 198.0 MeV beams, drifted from initial mean values of 0.0% and –0.5%, to –0.3% and –0.1% after Upgrade 2, respectively. For carbon ions, the standard dose deviation remained within 0.3%–0.4% during the course of the Upgrades. The mean dose deviations, however, were significantly affected after Upgrade 2 as a function of energy: the 120.0 and 346.6 MeV/n beams, drifted from initial mean values of 0.6% and 0.8% to –0.5% and +1.1%, respectively. The Upgrade 2 had therefore a major effect on the dose output spread as a function of energy, between the highest and the lowest energies. The only way to improve such deviations is to make a new calibration of the beam monitoring system^17^ in number of particles per count, by adapting the energy‐dependent dose output calibration factors. However, due to limitations in the precision of the polynomial fits used in the beam monitors for this purpose and the number of available energies, no significant improvement was provided by the definition of new calibration factors, thus the dose output calibration was not modified. The exact reason behind such behavior (the dose output spread as a function of energy) is currently unknown, even if potential root causes have been identified. The dose output is a sensitive parameter. Indeed, experience showed us that fluctuations in beam intensities at the source/synchrotron level are correlated with beam size and dose variations. The beam monitors are also sensitive to rapid variations in the temperatures in winter and summer time (seasoning effects) and are usually a root cause for energy‐independent dose drifts, which are corrected by the application of a scaling factor (QAKfit) within ±3%, typically. Since proton beams for the same IR2H were not affected by the Upgrade 2, it is rather unlikely that the observed variations for carbon ions are due to the beam monitors and therefore variations may most likely be due to differences in the delivered carbon ion beam itself. One potential root cause to explain the energy‐dependent dose output spread for carbon ions after Upgrade 2 may be the spray radiations from the nozzle. Indeed, variations in the spectra of spray radiation (as defined in Ref. [18]) and/or variations in the beam optics of secondaries may induce a different dose‐response ratio between the reference ionization chamber placed at isocenter and the beam monitors in the nozzle. This potentially leads to an energy‐dependent dose output spreading.

**TABLE 3:** Statistical evaluation of dose output deviations compared to the baseline value, considering the initial machine and the upgraded machine configurations (Upgrades 1 and 2). All values are provided as percentage

	Protons	62.4 MeV	97.4 MeV	148.2 MeV	198.0 MeV
Initial machine	mean value	0.0	–0.2	0.2	–0.5
	std dev	0.3	0.3	0.3	0.3
Upgrade 1	mean value	0.0	0.1	0.1	–0.5
	std dev	0.2	0.3	0.3	0.3
Upgrade 2	mean value	–0.3	0.0	0.3	–0.1
	std dev	0.3	0.3	0.4	0.4

	Carbon ions	120.0 MeV/n	213.4 MeV/n	284.7 MeV/n	346.6 MeV/n
Initial machine	mean value	0.6	0.3	0.6	0.8
	std dev	0.4	0.4	0.4	0.4
Upgrade 1	mean value	0.2	–0.2	–0.3	0.2
	std dev	0.4	0.3	0.3	0.3
Upgrade 2	mean value	–0.5	0.7	0.4	1.1
	std dev	0.4	0.4	0.4	0.4

### 3.6 QA tolerances and action levels

The uncertainty of the spot sizes and absolute spot positions measured by Lynx were evaluated as 0.2 and 0.3 mm, respectively.^3^ The detailed characterization of Lynx for protons and carbon ions as reported by CNAO^9^ is compatible with our previous estimates,^3^ even if uncertainty budgets were not provided.^9^ Using the Sphinx/Lynx, the spot position uncertainty is assumed to be a combination of image registration and patient positioning uncertainty (estimated to 0.5 mm for our Sphinx set‐up) and absolute position measurement uncertainty from Lynx of 0.3 mm,^3^ leading to a total combined uncertainty of 0.6 mm. The reproducibility of proximal and distal range parameters was found to increase with energy and were up to 0.14 mm for the maximum proton energy of 200 MeV.^8^ These results are consistent with our range parameter reproducibility for protons of about 0.1 mm (Table 1), even if for carbon ions larger reproducibility values have been observed for the Bragg peak width (up to 0.4 mm), mainly due to proximal range measurement uncertainties. Our action levels are split between warning levels and fail levels. A warning level requires the user to evaluate the QA trendlines and plan appropriate corrective actions as soon as possible. A fail level prevents patient treatment and a corrective action must be implemented immediately. The impact of beam range and position uncertainties are rather straightforward to understand, as they directly influence the 3D positioning accuracy of each spot in the patient. The most difficult tolerance to define is the spot size. The impact of 10%, 25% and 50% spot size variations for proton and carbon ions were considered as typical fluctuations, worst‐case scenario and fault conditions, respectively.^19^ While 10% variations were found negligible, variations up to 25% had clinical impact ranging from negligible to moderate. Based on all the considerations discussed in this section, our QA warning and fail levels were established (Table 4).

**TABLE 4:** MedAustron daily QA warning and fail levels for protons and carbon ions

		Protons		Carbon ions	
Sphinx/Lynx region	QA parameter	Warning	Fail	Warning	Fail
Spot	Beam position (x,y)	1.5 mm	3 mm	1.5 mm	3 mm
	Beam size (x,y)	20%/2 mm	40%/3 mm	20%/2 mm	40%/3 mm
Bragg peak	Distal range	1 mm	2 mm	1 mm	2 mm
	Proximal range	1 mm	2 mm	1 mm	2 mm
	Width	1 mm	2 mm	1 mm	2 mm
	Fall‐off	1 mm	2 mm	1 mm	2 mm
Central fiducial	Coincidence (x,y)	1.5 mm	3 mm		
Homogeneity	Homogeneity (1D)	3%	6%	3%	6%
Dose	Dose	2%	3%	2%	3%

One should note that AAPM only refers to tolerances and does not explicitly state which action to take in case of deviation. In the following, AAPM tolerances are compared to our action levels. For ranges, AAPM recommends 1 mm, while we consider 1 mm as warning level. For spot sizes, 10% is recommended. It is actually an average spot size, while in our case we evaluate the spot size in x and y separately. Our warning level is set to 20%, based on machine drifts observed along the various upgrades (Figure 6) and clinical recommendations.^19^ For spot positions, the AAPM recommends 2/1 mm for absolute and relative positions. We only consider absolute positions with a warning level set at 1.5 mm (our position warning/fail levels account for daily set‐up and image registration uncertainties. They are reduced to 1/2 mm for monthly QA procedures using alignment based on room lasers). The recommended AAPM tolerance for homogeneity is 2% against the reference value obtained during commissioning. We use instead an absolute homogeneity threshold set to 3% as warning level. The recommended AAPM tolerance on dose is 3%, while we use 2% as warning level. In addition to the set tolerances and action levels, a regular review of the QA trendlines allows identifying machine drifts even before reaching QA thresholds. Therefore, not only the definition of the QA tolerances and action levels is important, but also the follow‐up of QA trendlines.

### 3.7 Performances of the QA process

The daily QA implemented at MedAustron encompasses beam delivery QA and in‐room equipment QA (treatment couch with integrated imaging system). Despite the details of the in‐room QA being out of the scope of this article, the performances reported correspond to the entire QA workflow (beam delivery and in‐room equipment QA) over the time‐frame 17/06/2019‐1/12/2021, when Sphinx/Lynx was implemented for protons and carbon ions. The entire daily QA is performed in 2 rooms in parallel in less than 2 h. The two rooms include three proton beam lines (2 horizontal and 1 vertical) and two carbon ion beam lines (horizontal and vertical). The average QA time per beamline is about 20 min only. The beam delivery QA time includes Sphinx/Lynx set‐up, full data analysis and QA approval in the myQA software for more than 70 beam delivery parameters per beamline. The Sphinx/Lynx daily QA set‐up allows for integrated daily QA tests and provides a quasi‐end‐to‐end test, as the registration process of Sphinx is performed against a reference CT image acquired during Sphinx commissioning. On a monthly basis, different equipment and set‐ups are used to specifically check the beam size and position (another Lynx), range (Giraffe, IBA‐dosimetry, Schwarzenbruck, Germany) and dose (ROOS ionization chamber in RW3 slabs, PTW, Freiburg, Germany) in the room coordinates (using room lasers for alignment). This equipment is therefore independent of image registration and specific to each beam delivery parameter. It allows for independent and specific checks of the beam delivery parameters. The monthly QA equipment can be used in case of unexpected daily QA deviation, to verify specific beam parameters. One should note in addition that a QA program of the QA equipment was implemented.^3^ This QA program allows monitoring the performances of the QA equipment and it includes, among other tests, cross‐checks of beam size, position, range and dose between daily and monthly QA.

## 4 CONCLUSION

This paper presented the first implementation of the Sphinx/Lynx device (including the Advanced Markus ionization chamber) for comprehensive daily QA of a horizontal proton and carbon ion beam line. The data included the review of QA trendlines for more than 3 years for protons and more than 2 years for carbon ions. It allowed to identify specific changes in the beam delivery parameters and their standard deviation due to upgrades made to the machine configuration. The Sphinx/Lynx system was found to be a useful and efficient integrated device to serve the purpose of daily QA for dual particle facilities. The definition of tolerance and action levels as currently applied at MedAustron was presented and discussed in light of existing literature. While some differences are observed they are in general in agreement with the literature and in line with recommendations provided in AAPM Report TG‐224. The full daily QA workflow, including QA approval of more than 70 beam delivery parameters per beamline, is performed in about 20 min per beamline on average for the described implementation.

## AUTHOR CONTRIBUTIONS

Loïc Grevillot: First author and main investigator of the study. Jhonnatan Osorio Moreno, Hermann Fuchs, Ralf Dreindl, Alessio Elia, Marta Bolsa‐Ferruz: participated in data acquisition, trendline analysis and manuscript review. Markus Stock and Hugo Palmans: Senior authors supervising the work.

## CONFLICT OF INTEREST

The author declares no conflict of interest.

## Data Availability

The data that support the findings of this study may be requested from the corresponding author. The data are not publicly available due to privacy restrictions.
